# Multiple myomectomy to aid fertility treatment - surgical and fertility outcomes: a retrospective cohort study

**Published:** 2021-01-08

**Authors:** YE Şükür, E Saridogan

**Affiliations:** University College London Hospital, London, United Kingdom; Current address: Ankara University School of Medicine, Department of Obstetrics and Gynaecology, Ankara, Turkey.

**Keywords:** endometrium, hysteroscopy, infertility, in vitro fertilisation, intrauterine adhesion, multiple myomectomy

## Abstract

**Objective:**

To assess the effects of multiple myomectomy at laparotomy on fertility potential of infertile women who are planning to undergo assisted reproductive technology (ART) treatment.

**Methods:**

A retrospective single centre cohort study was conducted. Data of infertile women who were planning to undergo ART and underwent open myomectomy for multiple fibroids between January 2010 and December 2018 were reviewed. Data were collected on demographics, presenting symptoms, preoperative imaging findings, operative details, and postoperative and IVF outcomes. The primary outcome measure was the necessity for further hysteroscopic surgery prior to subsequent in vitro fertilisation (IVF) cycle. The secondary outcome measure was live birth rate.

**Results:**

A total of 55 women were included in the analyses. The median number of fibroids removed was 12 (range, 3-51). Thirteen (26%) women required further surgery before embryo transfer. In women with a breached endometrial cavity, the likelihood of further hysteroscopic division of intrauterine adhesions was increased 18-fold (P=0.017). Thirty-two women underwent 45 IVF cycles, 16 of which resulted in live birth (50%).

**Conclusions:**

Infertile women with large and multiple fibroids could have good fertility outcomes following open myomectomy. Special care must be taken not to damage the uterine cavity. The intraoperative breach of the endometrial cavity seems to increase the risk of intrauterine adhesions, which may further compromise ART treatment and outcomes. Hysteroscopy prior to any embryo transfer cycle may help to optimise the endometrial cavity and fertility outcome in women who underwent multiple myomectomy.

## Introduction

The impact of fibroids on fertility and fertility treatment outcomes has long been a matter of debate (Somigliana et al., 2007; Klatsky et al., 2008; Pritts et al., 2009). Uterine fibroids are frequently seen in women who undergo infertility treatment. The evidence currently available suggests a detrimental impact of submucosal fibroids (Klatsky et al., 2008; Pritts et al., 2009). There is also some evidence that intramural fibroids, that do not distort the uterine cavity, reduce the chance of success of in vitro fertilisation (IVF) treatment (Somigliana et al., 2007; Oliveira et al., 2004; Yan et al., 2014). One of the major issues is the size of the fibroids. A significant negative impact on fertility outcomes is suggested when the fibroid is larger than 3-4 cm (Oliveira et al., 2004; Yan et al., 2014).

Treatment options for women with uterine fibroids and who do not desire fertility include medical therapies along with hysteroscopic or abdominal removal surgeries. Those women, except with submucosal fibroids, could be offered oestrogen- progestin or progestin only contraceptives, progestin releasing intrauterine devices, GnRH analogues, uterine artery embolisation, focused ultrasound surgery, and hysterectomy, taking into consideration the age and symptoms of the patient. However, most medical therapies preclude conception, have adverse effects in the long term, and result in the return of symptoms after discontinuation of treatment. Hence, they are not appropriate for women who desire fertility. While uterine artery embolisation and focused ultrasound surgery are subject to concerns due to a loss of uterine blood supply, a lower ovarian reserve or placentation problems, myomectomy seems to be the best choice for women who desire fertility (Gupta et al., 2014; Rabinovici et al., 2010).

There is relatively little information on the safety of multiple myomectomy for fibroids that enlarge the uterus significantly in infertile women. Myomectomy in the presence of multiple fibroids can be associated with significant morbidity and may cause complications which may be detrimental to the assisted reproductive technology (ART) outcome. In this article we analysed the outcomes of women who underwent myomectomy at laparotomy for multiple and/or large fibroids in advance of ART or embryo transfer.

## Material and methods

### 

In this single centre retrospective cohort study, infertile women who underwent open myomectomy for multiple fibroids under the care of the same clinician (ES) between January 2010 and December 2018 were reviewed. Cases were identified from the clinical notes and operating room records. Data were collected on patient demographics (age, parity and previous miscarriage history, previous uterine surgery, and ART treatment history), presenting symptoms, preoperative imaging findings (number, size, and location of fibroids), operative details (number and size of fibroids removed, number and size of submucosal fibroids removed, estimated blood loss, hysteroscopic findings when performed, and if breach of the endometrial cavity occurred), postoperative outcomes (duration of hospital stay, complications, necessity for further surgery before next ART cycle), and ART cycle outcome if performed. All women underwent clinical examination and preoperative ultrasound and/ or magnetic resonance imaging. Miscarriage was defined as a non-viable intrauterine pregnancy up to 20 weeks gestation.

The inclusion criteria were infertile women who have an enlarged uterus due to multiple fibroids and an IVF treatment plan following open myomectomy. Exclusion criteria were the absence of infertility history, single or laparoscopic myomectomy and spontaneous conception after surgery. The uterine size at pelvic examination is presented as enlargement equivalent to weeks of gestation, which was 12 weeks when palpable out of the pelvis and 20 weeks when palpable at the level of umbilicus.

The primary outcome measure was intra-/post- operative complication rates and the secondary outcome measure was fertility outcomes of the women who tried for pregnancy. Estimated blood loss was calculated by subtracting the irrigation fluid volume from the total volume of fluids collected in the aspiration bag. Postoperative complications were evaluated according to Clavien-Dindo classification (Dindo et al., 2004). The postoperative complications recognised during hospital stay and during early follow-up were recorded.

All open myomectomies were performed using a similar technique under general anaesthesia following urethral catheterization. Diluted synthetic vasopressin (20 IU/20 mL saline) was injected into the myometrium as standard practice for all cases. Incisions to remove myomas were planned according to the location and size of the fibroids. Mechanical instruments were used to enucleate fibroids. If the endometrial cavity was opened, it was repaired using monofilament absorbable sutures. The myometrium was sutured with No 0 or No 1 absorbable polyglactin 910 sutures. The serosa was closed separately with a No 2-0 absorbable polyglactin 910 suture, and an anti-adhesion barrier was used in almost all cases. In all the operations, blood and irrigation fluid were aspirated and collected in suction containers, and the volume was measured using a graduated volume scale. In addition, the swabs and packs were weighed to calculate blood content. The total amount of blood loss was based on this objective measurement.

The postoperative monitoring of haemoglobin or haematocrit was not routine and was left to the lead surgeon’s discretion, depending on the blood loss during surgery, vital signs and the postoperative follow-up. The urinary catheter was removed on the first postoperative day at the earliest, and patients were discharged from day 2 onwards if they felt well and were able to empty their bladder. Before further IVF treatment, the endometrium was evaluated by ultrasonography and office hysteroscopy, particularly in cases of cavity breach at myomectomy. When intrauterine adhesions were detected, hysteroscopic division using hysteroscopic scissors was performed in the operating theatre under general anaesthesia. At least four weeks later, systematic second look hysteroscopy was performed before proceeding with the next IVF cycle.

### Statistical Methods

Data analyses were performed by using SPSS Version 21.0 (IBM Corporation, Armonk, NYC, USA). Samples were tested with a Kolmogorov-Smirnov test to determine the normality of distributions. According to the results, non-parametric tests were preferred. Continuous variables were compared with a Mann-Whitney U test and categorical variables were compared with a Chi-square test or a Fisher’s exact test where appropriate. Multivariate logistic regression analysis with a model building strategy was used to determine independent predictors of further hysteroscopic surgery for intrauterine adhesions. A P value of <0.05 was considered statistically significant.

### Ethical Approval

Ethical approval was sought, and it was deemed that full ethical approval was not required because the project was considered to be solely service evaluation. Such projects do not require ethical review by the National Health Service (NHS) or Social Care Research Ethics Committee or management permission through the NHS Research and Development office. Under these circumstances, there was no need to submit applications to the NHS Research Ethics Committee or NHS/Health and Social Care Research and Development office (www.hra.nhs.uk).

## Results

**Figure 1:**
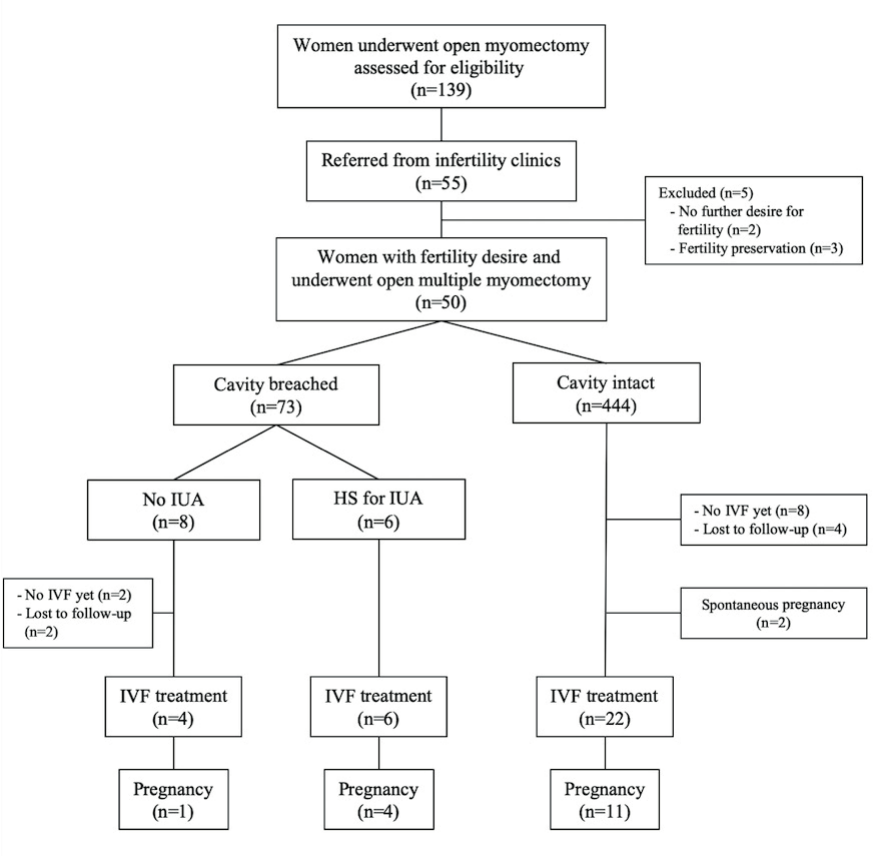
Flow-chart of the study population.

During the study period a total of 139 women underwent open myomectomy for multiple fibroids. Fifty-five (39.6%) of these women were referred from infertility clinics. Two women indicating that they would not continue IVF treatment and three women treated for fertility preservation were excluded from the final analyses. As a result, the data of 50 infertile women who underwent open myomectomy for multiple fibroids prior to IVF treatment were found to be eligible for this study. Figure 1 presents the flow- chart of the study population.

The median age of the study population was 38 years (range, 29-51 years). Four (8%) were parous and 46 had primary infertility. Twelve (24%) had at least one previous failed IVF cycle and 16 (32%) underwent an IVF cycle to freeze embryo/s prior to the surgery. 20 (40%) had pain, 15 (30%) had urinary frequency, 4 (8%) had bowel symptoms, 27 (54%) had heavy menstrual bleeding, and 12 (24%) had anaemia. Table 1 shows the demographics, the preoperative imaging and intraoperative findings. The median size of the uterus was 15 gestational weeks (range, 10-28) at preoperative evaluation. Apart from three women who had a previous mid- line incision scar, all cases were operated via a Pfannenstiel incision. The median number of fibroids removed was 12 (range, 3-51). No major intra- operative complications were recorded other than excessive blood loss. The endometrial cavities of 32 women (64%) were also evaluated by hysteroscopy just before open surgery (Table I). No intra-uterine adhesions were recorded at hysteroscopies.

**Table I:** Demographic characteristics and preoperative imaging and intraoperative findings.

N = 50	Median (min-max)	Mean±SD
Age, years	38 (29-51)	38.5±4.6
Parity no.	0 (0-1)	0.09±0.28
Previous miscarriage no.	0 (0-3)	0.4±0.7
Previous uterine surgery, n (%)	18 (36)	
Previous failed ART history, n (%)	12 (24)	
Women with frozen embryos, n (%)	16 (32)	
Preoperative evaluation		
Uterine size at examination, gestational weeks	15 (10-28)	16.3±4.1
Number of fibroids	6 (3-25)	8.2±6.3
Size of the largest fibroid, cm	8 (3-16)	7.9±2.8
Submucosal fibroid, n (%)	25 (50)	
Number of submucosal fibroids	1 (0-3)	0.9±1
Size of the largest submucosal fibroid, cm	3 (1-7)	3.1±2
Intra-operative findings		
Number of fibroids removed	12 (3-51)	15.5±12.2
Number of uterine incisions	3 (1-13)	3.4±2.3
Size of largest fibroid removed, cm	8 (3-15)	7.9±2.5
Breach of endometrial cavity, n (%)	14 (28)	
EBL, ml	250 (50-3000)	419±500
Number of women with EBL 3500 ml, n (%)	13 (26%)	
Hysteroscopy findings (N=32)		
Submucosal fibroid, n (%)	16 (50)	
Cavity distortion, n (%)	20 (62.5)	
Removal of fibroid, n (%)	4 (12.5)	
Polyp removal, n (%)	3 (9.4)	

Table II summarises the postoperative follow-up period of the study cohort. Six women (12%) who had postoperative intrauterine adhesions (IUA) underwent hysteroscopic division of IUA. Among those, five women underwent seven IVF cycles after hysteroscopic division of IUA and three pregnancies were achieved.

**Table II:** Postoperative follow-up period and IVF results.

N = 50		
Postoperative period		
Hospital stay, days		3 (2-10)
Postoperative complications*, n (%)		4 (8)
	- Pelvic haematoma	1 (2)
	- Loin pain	1 (2)
	- Non-specific fever	1 (2)
	- Constipation	1 (2)
Transfusion, n (%)		2 (4)
Further surgery, n (%)		13 (26)
	- Hysteroscopic division of IUA	6 (12)
	- Hysteroscopic removal of fibroid	6 (12)
	- Laparoscopic myomectomy	1 (2)
Pregnancy outcome		
Spontaneous pregnancy, n (%)		2 (4)
Embryo transfer, n (%)		
	Performed	32 (64)
	Not yet	10 (20)
	Unknown	6 (12)
Endometrial thickness, mm		9 (7-12.5)
Live birth among IVF patients, n (%)		16/32 (50)

*Note:* Values are presented as median (min-max) for continuous variables and as frequency (percentage) for categorical variables. *Clavien-Dindo class I-II. IUA: intrauterine adhesion; IVF: in vitro fertilization.

Two women conceived spontaneously with no need for IVF treatment, six women were lost to follow-up at IVF treatment stages, and ten women have not had any IVF cycles at the time of article preparation for publication. To date, thirty-two women underwent 45 IVF cycles, 16 of which resulted in live birth (Table II). Eleven women (22%) experienced at least one miscarriage episode. In a subgroup analysis when compared to the rest of the cohort, they had similar rate of previous intrauterine surgeries, uterine sizes, number of total and submucosal fibroids. However, the rate of women who underwent hysteroscopic division of IUA was significantly higher among women who had miscarriage history than in those who did not (36.4% vs. 5.1%, respectively; P=0.016).

Table III shows the comparison of women who required further hysteroscopic division of IUA and who did not. Previous uterine surgery, previously failed ART history, and breach of the endometrial cavity at open myomectomy were significantly higher in the further hysteroscopic IUA surgery group. Multivariate logistic regression analysis revealed that a breach of the endometrial cavity at open myomectomy significantly increased the likelihood of further hysteroscopic division of IUA, when adjusted for age, miscarriage history, previous uterine surgery and previous failed ART history (OR: 22.595, 95% confidence interval 1.948-262.136; P=0.013) (Table IV).

**Table III:** Comparison of women who required further hysteroscopic division of intrauterine adhesions and those who did not.

	HS-IUA N=6	No adhesion N=44	P
Age, years	42.5 (34-51)	38 (29-48)	0.160
Previous miscarriage no.	1 (0-3)	0 (0-2)	0.061
Previous uterine surgery, n (%)	5 (83.3)	13 (29.5)	0.020
Previous failed ART history, n (%)	4 (66.7)	8 (18.2)	0.026
Preoperative uterine size, weeks	13 (10-18)	16 (10-28)	0.084
Number of fibroids removed	11 (3-52)	12 (3-51)	0.704
Number of uterine incisions	2 (1-5)	3 (1-13)	0.305
Size of largest fibroid removed, cm	6.5 (4-13)	8 (3-15)	0.594
Breach of endometrial cavity, n (%)	5 (83.3)	8 (18.2)	0.005
Estimated blood loss, ml	300 (50-500)	275 (100-3000)	0.381
Postoperative complication, n (%)	0 (0)	4 (9.1)	0.430

*Note:* Values are presented as median (min-max) for continuous variables and as frequency (percentage) for categorical variables. HS-IUA: intrauterine adhesion at hysteroscopy; ART: assisted reproductive technology.

**Table IV:** Multivariate logistic regression analysis demonstrating predictors of further hysteroscopic division of IUA before IVF.

	OR	95% confidence interval (CI)	P
Previous miscarriage history	7.622	0.864-67.253	0.068
Breach of endometrial cavity	22.595	1.948-262.136	0.013

*Note:* Analyses are adjusted for age, previous uterine surgery, previous failed ART history, miscarriage, and breach of endometrial cavity. Variables in the equation at last step are presented. ART: assisted reproductive technology.

## Discussion

The present study was conducted to assess the outcomes of women who underwent myomectomy at laparotomy for multiple and/or large fibroids in advance of ART or embryo transfer. According to the results obtained from our study, a significant proportion of infertile women with multiple fibroids conceive and deliver following a successful operation. A non-negligible part of those require further surgery due to intrauterine adhesions prior to ART. The most significant risk factor for intrauterine adhesions is a breach of the endometrial cavity.

Significant fibroids are found in 10-15% of white women and 30-40% of black women in reproductive years, and they are more frequent in infertile women (Baird et al., 2003). Uterine fibroids may have a negative impact on fertility. There is a definite consensus on the adverse effects of submucosal fibroids on fertility and the outcome of fertility treatments (Somigliana et al., 2007; Pritts et al., 2009; Eldar-Geva et al., 1998; Varasteh et al., 1999). Submucosal fibroids anatomically distort the endometrial cavity and alter the intracavitary environment. Similar mechanisms may apply for the intramural fibroids that distort the endometrial cavity. Most clinicians agree that the removal of submucosal fibroids before any fertility treatment is beneficial (Klatsky et al., 2008; Kroon et al., 2011; Saridogan & Saridogan, 2019).

However, many intramural fibroids have no endometrial cavity distortion, the effect of which on fertility and outcome of fertility treatment remains uncertain. The plausible mechanisms proposed as factors associated with reduced fertility potential for non-cavity distorting fibroids are functional changes in the endometrium and myometrium, and endocrine and paracrine changes that may alter the uterine environment and negatively affect implantation (Zepiridis et al., 2016). A previous study evaluating IVF cycle results of more than 10.000 women failed to show an impact of fibroids that do not distort the cavity (Yan et al., 2014). This finding was supported by some other studies (Klatsky et al., 2008; Somigliana et al., 2011). However, all of those studies included subserosal fibroids too. In contrast, several other studies suggested a negative impact of intramural fibroids that do not distort the cavity on the ART outcome (Khalaf et al., 2006; Surrey et al., 2001; Sunkara et al., 2010; Behbehani et al., 2018; Christopoulos et al., 2017). Although different cut-off levels have been identified in different studies, a recent comprehensive analysis found that the deleterious effect of fibroids on live birth rates was significant in women with two or more fibroids and in women with fibroids <3 cm (Christopoulos et al., 2017). Single fibroids with a dimension of <3 cm seem to have no negative impact on ART outcome (Christopoulos et al., 2017).

Overall, submucosal and intramural fibroids seem to have a negative impact on fertility. Hence, we can assume that multiple fibroids have significant adverse effects on the outcome of fertility treatments. Most of the studies in the literature exclude women with multiple or very large fibroids while reporting the effects of fibroids on fertility treatment outcome. Those women usually undergo myomectomy instead of proceeding with ART. The cohort in our study consisted of a special and somehow unreported group of patients. To the best of our knowledge, this is the first study to report the outcome of open multiple myomectomy prior to ART cycle in infertile women. According to our results, the cumulative live birth rate was 50% among those who completed IVF, which might be accepted as a good outcome in this group of women with relatively large uteri. In addition, a small proportion of patients conceived spontaneously before an ART attempt which could support a positive effect of fibroid removal.

Although the published literature suggests a possible detrimental impact of submucosal fibroids and intramural fibroids that distort uterine cavity, evidence of the benefits of myomectomy for both these fibroid types is still lacking. There is also a paucity of information on the removal of fibroids that do not distort the cavity prior to ART. Submucosal fibroids are usually dealt with via the hysteroscopic route. When an abdominal approach is required, the laparoscopic approach has the benefit of reduced pain, a shorter hospital stay and a quicker recovery (Bhave Chittawar et al., 2014). However, the laparoscopic approach takes longer to perform compared to an open myomectomy. The case for an open myomectomy is probably stronger in the presence of multiple fibroids which may take much longer to remove at laparoscopy. In addition, locating and removing smaller fibroids in the presence of multiple fibroids may be more difficult and this may potentially lead to persistent or recurrent fibroids. For this reason, reproductive surgeons may choose the laparotomy option beyond a certain threshold based on the number and size of fibroids or overall size of the uterus. In our practice, we perform laparoscopic myomectomy for the majority of women who need to undergo an abdominal fibroid removal operation (Bean et al., 2017). However, an open approach is preferred in the presence of multiple fibroids, some of which may be relatively small, in unusually large fibroids and when the woman declines a laparoscopic operation.

Potential complications of an open myomectomy for multiple fibroids include intraoperative bleeding, the need for transfusion, injury to other organs, infection, and pain (Afolabi et al., 2019). In our study we did not identify any intraoperative complications, other than excessive bleeding. A significant number of women (n=13, 26%) had intraoperative bleeding of more than 500 ml. Furthermore, 3000 ml bleeding was noted in one, and another woman suffered from a postoperative pelvic haematoma which resolved spontaneously. In addition, two women required blood transfusions. Although multiple myomectomy is quite successful in terms of fertility potential, intraoperative bleeding and risk of complications should be considered and discussed with patients before the operation.

Longer term complications of multiple myomectomy include intraabdominal and intrauterine adhesions which could potentially compromise ART outcome. According to our results, 12% of all infertile women undergoing multiple myomectomy would have IUA. Possible risk factors for IUA are previous intrauterine surgeries and a breach of the cavity during myomectomy. We identified an 18 times increased risk of IUA following a breach of the cavity during multiple myomectomy. This significant risk makes it necessary to carefully evaluate the uterine cavity of infertile women who underwent multiple myomectomy prior to ART treatment. Hysteroscopic evaluation of the uterine cavity would be sensible before further ART if the cavity was breached during multiple myomectomy. Among our study population the median time for hysteroscopic removal of IUA was 5 months which suggests earliest evaluation prior to embryo transfer considering the possibility of recurrent interventions.

Despite the small sample size and retrospective nature of the analyses, that we recognize as the main limitations of our study, the systematic exploration of individual parameters including pre- operative and intra-operative findings as well as fertility treatment outcomes, and the multivariate approach may add credence to our observations. In addition, ours is the first study reporting the surgical and fertility outcome of a relatively rare subgroup of infertile women. Other limitations of the study noted were the absence of a control group and the absence of data on fertility potential of women regarding ovarian reserve. However, considering the potential negative effects of not treating such a condition and the retrospective design of the study it was not possible to compare the results with a control group.

In conclusion, infertile women with large and multiple fibroids could have good fertility outcomes following open myomectomy. Special care must be taken not to damage the uterine cavity. The intraoperative breach of the endometrial cavity seems to increase the risk of intrauterine adhesions, which may further compromise ART treatment and outcomes. Hysteroscopy prior to any embryo transfer cycle may help to optimise the endometrial cavity and fertility outcome in women who underwent multiple myomectomy.
